# Supplementary material for: Human GBP1 Is Involved in the Repair of Damaged Phagosomes/Endolysosomes
Source: Int J Mol Sci. 2023 Jun 2;24(11):9701. doi: 10.3390/ijms24119701 (PMC10253393; doi:10.3390/ijms24119701)
Supplement: Supplementary file 1 [file ijms-24-09701-s001.zip › ijms-2236111-supplementary.pdf]

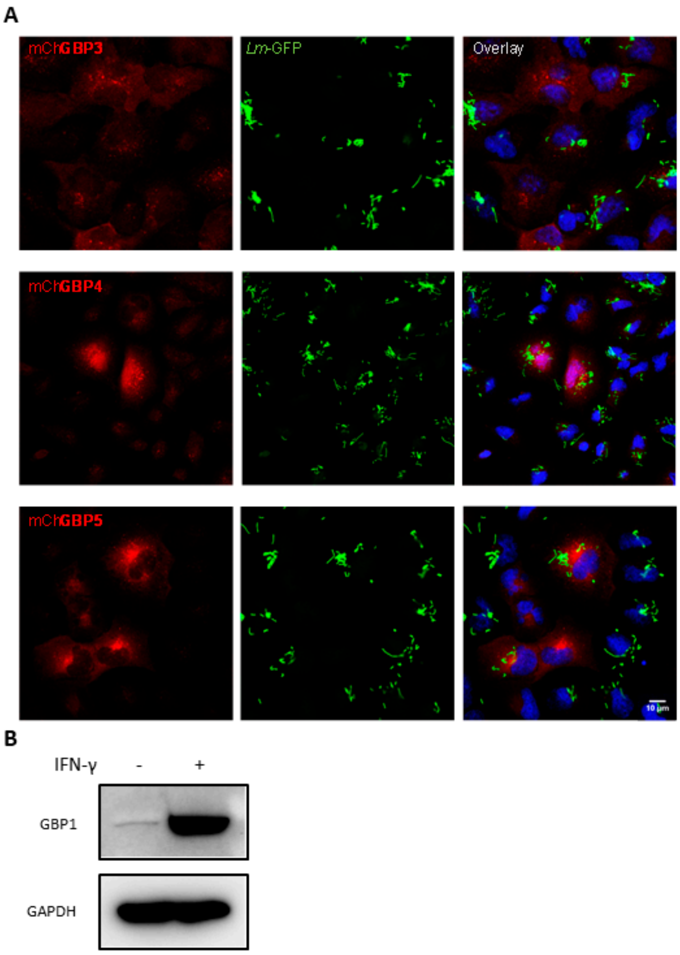

Supplementary Figure S1: (A) hGBP3, hGBP4 or hGBP5 are not recruited to intracellular *L. monocytogenes*. A549 cells expressing mCherry-hGBP3, hGBP4 or hGBP5 were infected with *L. monocytogenes* GFP (Lm-GFP) for 1h. Scale Bar: 10μm. Representative images from 3 independent experiments are shown. (B) Western blotting analysis of hGBP1 expression levels. A549 cells were treated with or without IFN-γ (1000 U/mL) overnight and the cell lysates were collected for SDS-PAGE and blotted with the specific anti-GBP1 antibody.

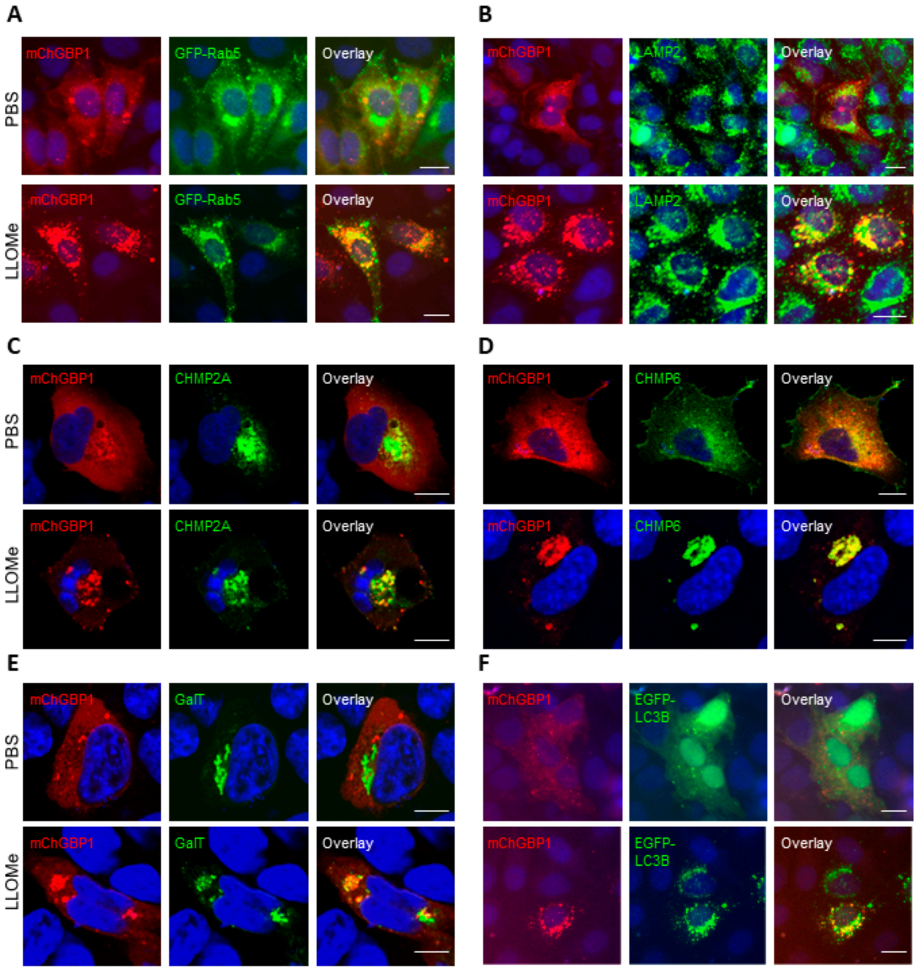

Supplementary Figure S2: hGBP1 co-localization with GFP-Rab5 (**A**), LAMP2 (**B**), CHMP2A (**C**), CHMP6 (**D**), GalT (**E**) or EGFP-LC3B (**F**). (**A, C-F**) A549 cells expressing mCherry-hGBP1 were transfected with GFP-Rab5 (**A**), CHMP2A-GFP (**C**), CHMP6-GFP(**D**), GalT (**E**) or EGFP-LC3B (**F**) overnight and then treated with PBS or LLOMe for 1 h. (**B**) A549 cells expressing mCherry-hGBP1 were mock-treated (PBS) or treated with LLOMe (1 mM) for 1 h and stained with antibodies against LAMP2.

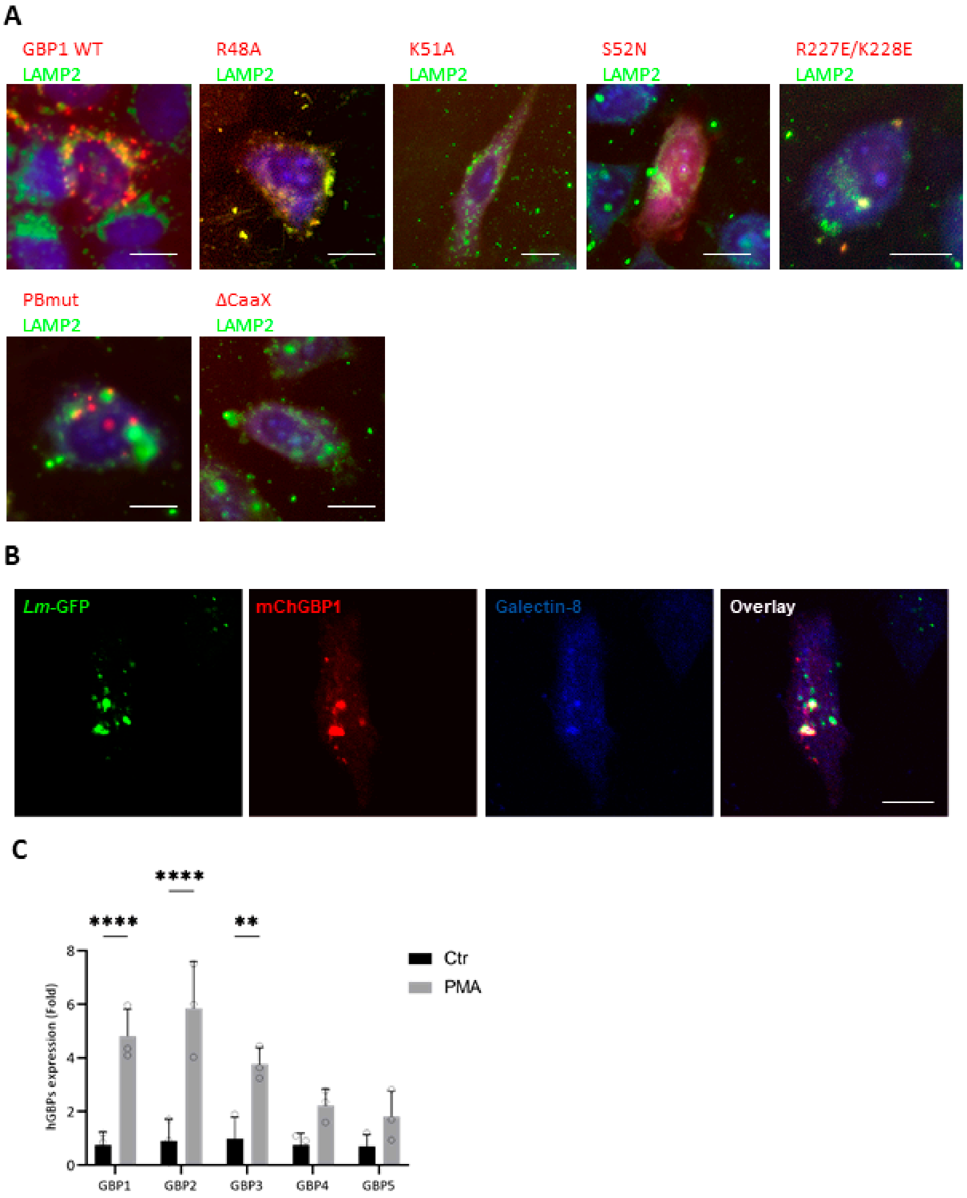

Supplementary Figure S3: (A) The colocalization of mCherry-hGBP1 mutants with LAMP2. A549 cells were transfected with mCherry-hGBP1 mutants, then treated with LLOMe for 1 h and stained with the antibody against LAMP2. (B) The colocalization of mCherry-hGBP1 with Galectin-8 during *L. monocytogenes* infection. A549 cells expressing mCherry-hGBP1 were infected with *L. monocytogenes* for 2 hours and stained with the antibody against Galectin-8. Scale bar: 20μm. (C) The relative expression levels of GBPs in THP-1 cells upon PMA stimulation. THP-1 cells were differentiated in the presence or absence of PMA (50ng/ml) for 24 hours and mRNAs were extracted for qPCR analysis. The relative expression levels were normalized to corresponding controls. Means±SD of 3 independent experiments.

Each dot represents one independent experiment. *P* values were calculated using two-way ANOVA. (**) p ⩽ 0.01, (****) p ⩽ 0.0001.

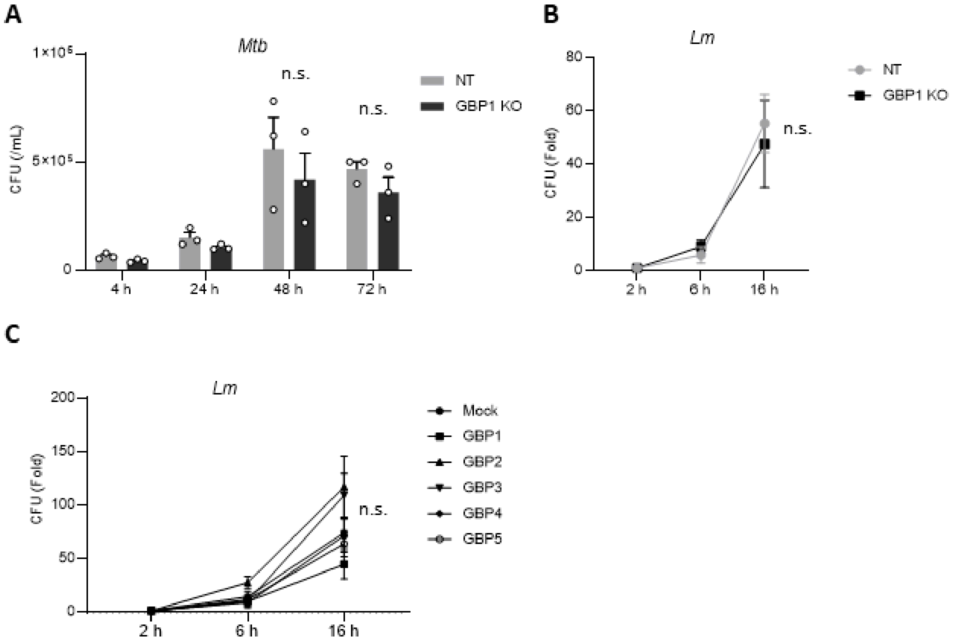

Supplementary Figure S4. hGBP1 does not restrict the control of *M. tuberculosis* and *L. monocytogenes* intracellular replication. Colony-forming unit (CFU) assay of intracellular growth of *M. tuberculosis* (Mtb) (A) or *L. monocytogenes* (Lm) (B, C). (A, B) THP-1 non-targeting control cells (NT) and hGBP1 KO cells were infected with Mtb or Lm for various time points. (C) A549 cells were transfected with empty plasmid (Mock) or individual hGBPs overnight, then infected with Lm for various time points. Data are shown as mean ± SD of 3 independent experiments. *P* values were calculated using two-way ANOVA. n.s. indicate non-significance.
